# Efficient Low-Cost Procedure for Microextraction of Estrogen from Environmental Water Using Magnetic Ionic Liquids

**DOI:** 10.3390/molecules26010032

**Published:** 2020-12-23

**Authors:** Paula Berton, Noureen Siraj, Susmita Das, Sergio de Rooy, Rodolfo G. Wuilloud, Isiah M. Warner

**Affiliations:** 1 Department of Chemistry, Louisiana State University, Baton Rouge, LA 70803, USA; nxsiraj@ualr.edu (N.S.); sdas@kol.amity.edu (S.D.); sergioderooy@yahoo.com (S.d.R.); iwarner@lsu.edu (I.M.W.); 2 Laboratorio de Química Analítica para Investigación y Desarrollo (QUIANID), Facultad de Ciencias Exactas y Naturales, Universidad Nacional de Cuyo/Instituto Interdisciplinario de Ciencias Básicas (ICB), CONICET UNCUYO, Padre J. Contreras 1300, Mendoza M5502JMA, Argentina; rodolfowuilloud@gmail.com; 3 Consejo Nacional de Investigaciones Científicas y Técnicas (CONICET), Buenos Aires C1425FQB, Argentina; 4 Chemical and Petroleum Engineering Department, University of Calgary, 2500 University Drive NW, Calgary, AB T2N 1N4, Canada; 5 Department of Chemistry, University of Arkansas at Little Rock, Little Rock, AR 72204, USA; 6 Department of Chemistry, Amity University Kolkata, Major Arterial Road, Action Area II, Kadampukur Village, Rajarhat, Newtown, West Bengal 700135, India; 7 Shell Oil Products US, 150 N Dairy Ashford Rd, Houston, TX 77079, USA

**Keywords:** magnetic ionic liquids, estrogens, microextraction, wastewater samples

## Abstract

In this study, three magnetic ionic liquids (MILs) were investigated for extraction of four estrogens, i.e., estrone (E1), estradiol (E2), estriol (E3), and ethinylestradiol (EE2), from environmental water. The cation trihexyl(tetradecyl)phosphonium ([P_66614_]^+^), selected to confer hydrophobicity to the resulting MIL, was combined with tetrachloroferrate(III), ferricyanide, and dysprosium thiocyanate to yield ([P_66614_][FeCl_4_]), ([P_66614_]_3_[Fe(CN)_6_]), and ([P_66614_]_5_[Dy(SCN)_8_]), respectively. After evaluation of various strategies to develop a liquid–liquid microextraction technique based on synthesized MILs, we placed the MILs onto a magnetic stir bar and used them as extracting solvents. After extraction, the MIL-enriched phase was dissolved in methanol and injected into an HPLC–UV for qualitative and quantitative analysis. An experimental design was used to simultaneously evaluate the effect of select variables and optimization of extraction conditions to maximize the recovery of the analytes. Under optimum conditions, limits of detection were in the range of 0.2 (for E3 and E2) and 0.5 μg L^−1^ (for E1), and calibration curves exhibited linearity in the range of 1–1000 μg L^−1^ with correlation coefficients higher than 0.998. The percent relative standard deviation (*RSD*) was below 5.0%. Finally, this method was used to determine concentration of estrogens in real lake and sewage water samples.

## 1. Introduction

Sample preparation is an important and usually mandatory step in analytical methodologies due to the complex properties of environmental matrices and low detection levels required by government regulations. Liquid–liquid extraction (LLE) is one of the most frequently used classical techniques for sample preparation. However, LLE is usually considered tedious and time-consuming since it requires multiple steps [1] and consumes toxic, organic solvents at high concentrations, thus generating large amounts of waste [2]. To overcome these problems, current trends in sample preparation are focused on use of miniaturized techniques, among which liquid–liquid microextraction (LLME) has attracted attention over the last few decades [3].

Ionic liquids (ILs), defined as salts with melting points below 100 °C [4], have become suitable alternatives to volatile organic solvents in extraction procedures due to their unique, tunable physicochemical properties, including low vapor pressure and thermal stability [5]. Among ILs, those based on trihexyl(tetradecyl)phosphonium chloride ([P_66614_]Cl) are thermally stable, and typically form liquid–liquid biphasic systems with an aqueous phase, making them suitable for use in extraction [6]. Furthermore, in comparison with ILs based on imidazolium or pyridinium, phosphonium-based ILs, and specifically [P_66614_]Cl are easier to synthesize, thus representing a relatively inexpensive IL on a ton-scale [7]. However, disadvantages of phosphonium-type ILs for LLME are attributed to their high viscosity, low density, and tendency to form difficult-to-separate emulsions, all of which complicate separation of phases and recovery of the analyte. To overcome these problems, researchers have proposed strategies including single drop microextraction and on-line based microextraction procedures [5,8,9,10].

An alternative method has been proposed by Deng et al., who used a non-dispersive solvent microextraction based on magnetic ILs (MILs) [11]. Since then, MILs containing a transition metal (e.g., iron, nickel, and cobalt) or rare-earth (e.g., dysprosium) ions that exhibit magnetic response [12] have been proposed for numerous analytical applications [13,14]. In particular, a combination of trihexyl(tetradecyl)phosphonium cation ([P_66614_]^+^) with magnetic anions aids in overcoming the formation of undesirable emulsification and avoids the centrifugation step since an external magnetic field can be used to separate and recover the MIL phase. Several phosphonium-based MILs were synthesized by combining [P_66614_]^+^ with tetrachloromanganate(II), tetrachloroferrate, tris(hexafluoroacetylaceto)manganate(II), tris(hexafluoroacetylaceto)nickelate(II), tris(hexafluoroacetylaceto)cobaltate(II), and tetrakis(hexafluoroacetylaceto)dysprosate(III). These phosphonium-based MILs were used in microextraction procedures for determination of pharmaceutical drugs, phenolics, insecticides, lipophilic organic UV filters, and polycyclic aromatic hydrocarbons in environmental waters [15,16,17,18]; cadmium and arsenic in honey [19,20]; estrogens in urine, milk, and cosmetics [21,22]; pesticides in vegetables [23]; and short-chain free fatty acids in milk samples [24].

In the present work, various MILs based on the cation [P_66614_]^+^ were synthesized, characterized, and evaluated as extraction solvents for separation and preconcentration of estrogens, before determination using HPLC-UV. Natural (estrone, E1; estradiol, E2; and estriol, E3) and synthetic (ethinylestradiol, EE2) estrogens are classified as potent endocrine-disrupting compounds, frequently found in natural and wastewater [25,26]. Their determination is, however, challenging due to complexity of the environmental samples and low concentrations of these analytes [27]. Hence, using magnetic properties of the MIL, we evaluated different microextraction strategies and optimized a microextraction technique to their determination in wastewater and tap and lake water samples.

## 2. Results and Discussion

**Figure 1:**
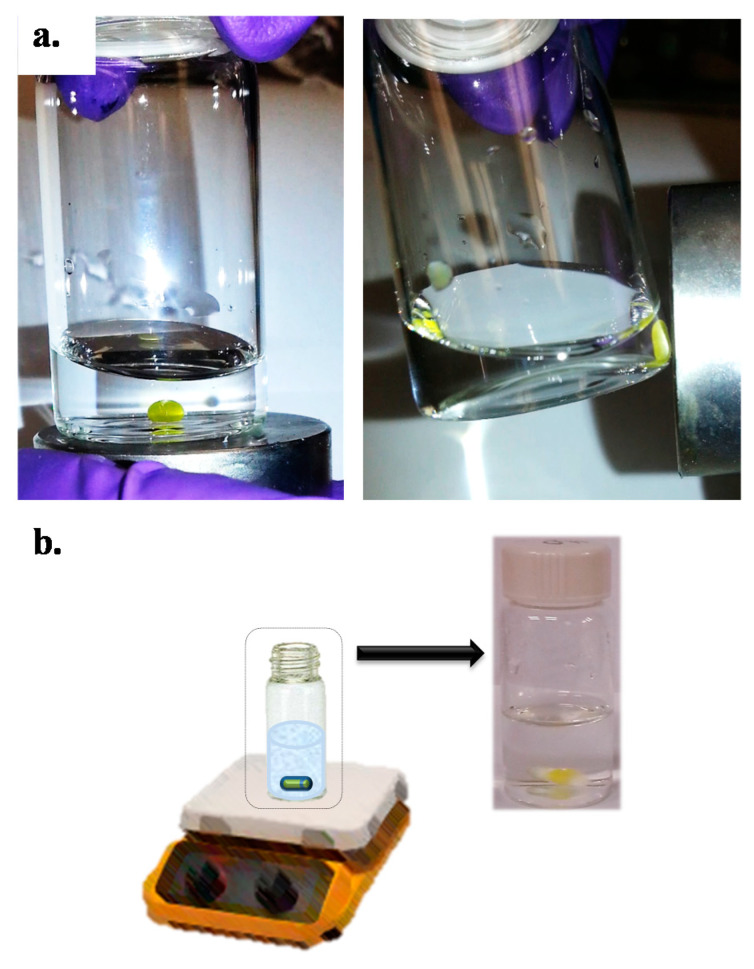
(**a**) Effect of an external magnetic field on the magnetic ionic liquid (MIL), and (**b**) schematic representation of final microextraction setup. The MIL (yellow) remained attached to the magnetic stir bar during extraction time.

The MILs based on [P_66614_]^+^ trihexyl(tetradecyl)phosphonium tetrachloroferrate(III) ([P_66614_][FeCl_4_]), trihexyl(tetradecyl)phosphonium ferricyanide ([P_66614_]_3_[Fe(CN)_6_]), and trihexyl(tetradecyl)phosphonium dysprosium thiocyanate ([P_66614_]_5_[Dy(SCN)_8_]) were synthesized and characterized as previously reported [11,28]. The magnetic properties of the resulting MILs were evidenced by their strong attraction to one tesla (1T) of an external magnetic field. As an example, the effect of the magnetic field on the MIL [P_66614_]_3_[Fe(CN)_6_] is shown in Figure 1a. The viscosities at room temperature of the synthesized MILs were 0.914, 6.63, and 2.96 Pa s for [P_66614_][FeCl_4_] [11], [P_66614_]_3_[Fe(CN)_6_], and [P_66614_]_5_[Dy(SCN)_8_], respectively.

Using the properties of high viscosity and magnetic susceptibility of the MILs, we evaluated various microextraction procedures to achieve the highest extraction recoveries of the analytes. Initially, the magnetic-based technique previously reported by our group for phenol extraction (technique #1) was tested [11]. In this procedure, the MIL was suspended in aqueous solution and moved synchronously with an external magnet by use of an orbital shaker [11]. In the second technique (technique #2), the MIL was also suspended in the aqueous solution, and the solution was stirred with a stir bar. For the third microextraction technique (technique #3), the MIL was placed onto a stir bar and the aqueous solution was then added (Figure 1b). The MIL remained attached to the stir bar during stirring due to its strong paramagnetism and high viscosity. This last extraction strategy was previously demonstrated for determination of lipophilic UV filters in waters [18].

**Figure 2:**
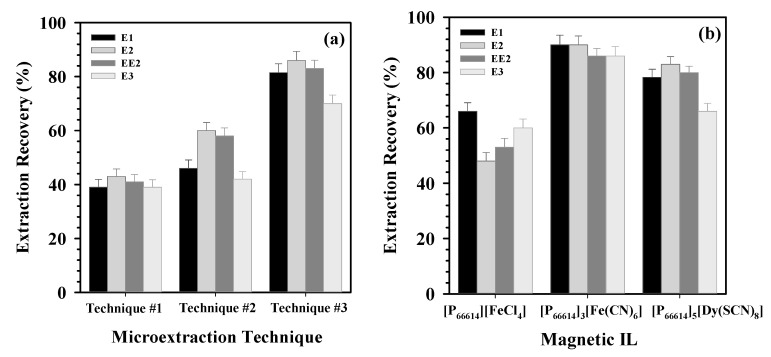
Effect of (**a**) different magnetic-based microextraction techniques and (**b**) different MILs on extraction recoveries of E1, E2, EE2, and E3, (*n* = 3). Other experimental conditions are included in Table 4.

The three techniques were evaluated for estrogen extraction using MIL [P_66614_]_3_[Fe(CN)_6_]. In all cases, the extraction time and amount of MIL were held constant. Best results were obtained using the third strategy (Figure 2a), i.e., when the MIL was attached to the stir bar before addition of the aqueous solution. Analyte extraction from the aqueous phase to the organic phase is facilitated by stirring, thus resulting in high extraction efficiency [29]. Furthermore, the extraction process was simplified, since no separation step, e.g., centrifugation, was required after extraction.

Using technique #3, the MILs [P_66614_][FeCl_4_], [P_66614_]_3_[Fe(CN)_6_], and [P_66614_]_5_[Dy(SCN)_8_] were then evaluated as extracting phases to achieve the highest extraction recoveries of analytes from the aqueous phase (Figure 2b). The highest recoveries for estrogens were observed using [P_66614_]_3_[Fe(CN)_6_] and [P_66614_]_5_[Dy(SCN)_8_] MILs. This difference in extraction may have been due to differences in the number of cations per mole of MIL. Even when the cation is the same for all evaluated MILs, an increase in the number of cations per molecule may increase the hydrophobicity, and hence the affinity of the analytes toward the MIL phase. As an example, the partition coefficients (K_o/w_) of [P_66614_]_3_[Fe(CN)_6_] and [P_66614_]_5_[Dy(SCN)_8_] were calculated as 6.105 (log K_o/w_ = 0.78) and as 34.5 (log K_o/w_ = 1.53), respectively, indicating a great influence of the number of cations on the hydrophobicity of these MILs. This trend was also observed using computational simulations, where an increase in the number of alkyl chains not only resulted in a higher distribution of organic compounds, but also in a decrease in solubility of the extracting phase in water [30]. The MIL [P_66614_]_3_[Fe(CN)_6_] was selected for further experimentation due to the lower price of its starting components and its simpler synthesis.

Once the microextraction procedure and MIL were selected, we used experimental design to identify and optimize variables that may influence recovery of analytes. In this study, on the basis of the literature and previous experiences in our group, we evaluated the effects of six factors, including MIL amount, sample volume, pH, salt addition, extraction time, and stirring rate. To simplify and reduce the number of experiments for optimization, we divided the design experiments into two steps: a fractional factorial design (FFD) followed by a central composite design (CCD). Systematic optimization procedures are performed to find the most important variables and to investigate the relationship between responses and variables by so-called response surface methodology (RSM) [31]. After optimizing responses, we used a multiple response criteria approach to simultaneously optimize the extraction recoveries of analytes E1, E2, EE2, and E3 into the MIL phase [32].

**Figure 3:**
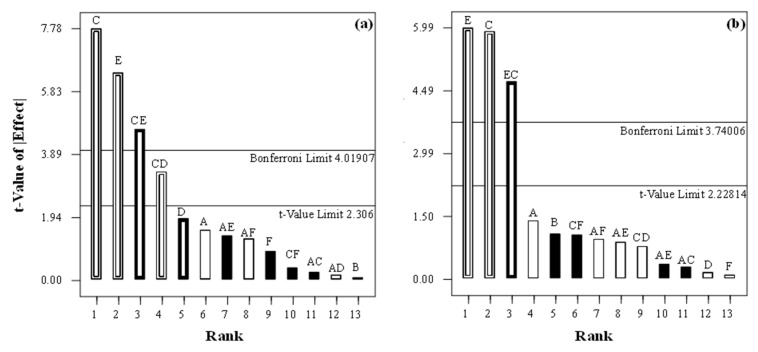
Example of Pareto charts of standardized effect on extraction recoveries of (**a**) E1 and (**b**) E2 for investigated analytes with different magnetic ILs.

As a screening step, an experimental FFD was built to determine the primary variables affecting recoveries of the analytes. The variables selected were MIL amount (20–45 mg), sample volume (4.0–15 mL), pH (4.0–8.5), salt addition (2.5–15.0% (*w*/*v*) NaCl), extraction time (15–45 min), and stirring rate (98–368 rpm). Values for each variable were chosen according to previous experiments. The evaluation process consisted of analyzing a water sample spiked at constant quantities of the analytes and for each variable combination suggested by the FFD, followed by determination of extraction recoveries in each case. After experiments, Pareto graphs were used to determine significant factors. An example of the results from the use of Pareto graphs is shown in Figure 3. Here, the bar height is proportional to the absolute value of the effect of each variable and can be used to compare its significance. Those variables, where effects were above the *t*-value and Bonferroni limits, were selected for optimization procedure, while other variables whose effects were below the *t*-value limit were considered to be insignificant. Shapiro–Wilk normality and ANOVA tests were used to confirm these results through evaluation of the normal distribution of the unselected variables. Individual variables that exhibited significant effects on analytical response (*p* < 0.1) of the analyzed estrogens were pH, salt addition, extraction time, and stirring rate, as well as their interactions.

It is well established that pH of a sample solution can significantly influence extraction recovery, particularly when acidic or basic solutes are extracted. As weak acidic compounds, estrogens exist in neutral form in acidic media and can be effectively extracted into MILs. Similar behavior has been reported for estrogen extraction using IL-dispersive LLME (DLLME) and stir bar sorptive extraction [33]. In a traditional LLE, the addition of salt may promote migration of organic compounds from the aqueous solution to the organic phase due to a salting-out effect, thus increasing the recovery. This effect was evidenced in the extraction recoveries of estrogens, which increased with addition of salt. Extraction time—which is defined as the period between the addition of aqueous sample and end of the stirring time—and stirring rate are crucial for diffusion of analytes and have a positive effect on recovery of the analytes. In contrast, neither the MIL amount nor the sample volume significantly influenced recoveries over the evaluated range. Since a minimum amount of MIL lowers consumption and warrants higher enrichment factors (increasing the volume ratio of donor to acceptor phase [34]), the highest phase ratio (i.e., 15 mL of aqueous solution and 26 mg of MIL) was selected for further experimentation.

A CCD was then applied to determine values of the four variables selected to achieve maximum recoveries of estrogens. This step consisted of 30 experiments based on a combination of the selected independent variables within the following ranges: (a) pH value: 4.0–8.5; (b) salt addition: 2.5–20 % (*w*/*v*) NaCl; (c) extraction time: 10–60 min; and (d) stirring rate: 60–570 rpm. As noted previously, both the MIL amount and sample volume were set according to results obtained in the screening phase (26 ± 1.0 mg and 15.0 mL, respectively). Experiments were performed in three blocks (three consecutive days) to remove expected variations caused by changes during experiments [31]. Outliers were removed by analyzing differences between fitted values test (DFFITS), which measures the influence that each point has on the predicted value, obtaining a standardized value interpreted as number of standard deviation units owed to experimental data that exerts disproportionate influence on the model [31]. The resulting behavior of the analytical response of analytes under the effect of the studied variables was best explained using linear (for EE2), 2-factor interactions (2FI) (for E2), and cubic models (for E1 and E4). The coefficients of each model were calculated using backward multiple regression and validated using ANOVA. Models were statistically significant (*p* < 0.05), and lack of fit was not significant (*p* > 0.05). *p*-values at 95% confidence level indicated that extraction time affected the extraction recoveries of all analytes and their analytical responses, while salt addition, pH, and stirring rate influenced the extraction recoveries of E1, E2, and E3.

**Figure 4:**
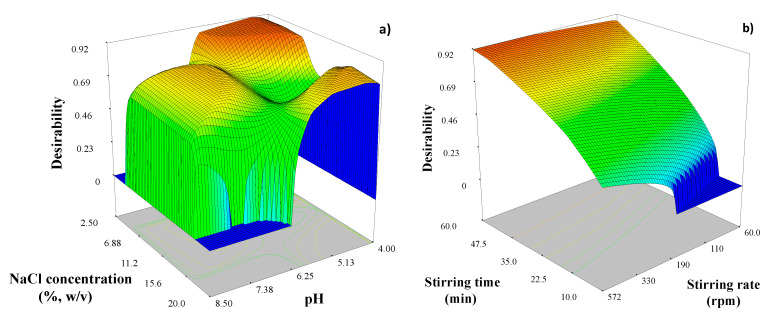
Response surface plots corresponding to the *D* function when optimizing (**a**) NaCl concentration vs. pH; (**b**) stirring time vs. stirring rate.

After modeling the responses, we used the desirability function (*D*) to find those extraction conditions that provide maximum extraction recoveries of all analytes. Following these conditions, we executed the optimization procedure and obtained the response surfaces for global *D* (Figure 4). Under the above-mentioned optimization criteria, the experimental conditions corresponding to one maximum in the desirability function (*D* = 0.901) were pH: 4.5; NaCl concentration: 6% (*w*/*v*); extraction time: 60 min; and stirring rate: 300 rpm. The suggested experimental values after optimization procedure were corroborated through a comparison between experimental and theoretical recoveries. No significant differences between predicted and experimental values were found.

The analytical figures of merit of the proposed methodology are summarized in Table 1. Calibration curves were determined using six working aqueous standards in the concentration range between 0.5 (for E3 and E2) and 1.0 (for EE2 and E1) and up to 1000 μg L^−1^ for analytes with correlation coefficients (*r*) higher than 0.9980. Limits of detection (LODs), based on signal at the intercept and three times the standard deviation of regression of the calibration curve, varied between 0.2 μg L^−1^ (E2 and E3) and 0.5 μg L^−1^ (E1). Reproducibility of the method was evaluated at 4 μg L^−1^ (*n* = 6) and expressed as relative standard deviation (*RSD*), with results in the range from 4.1% (E2) to 5.0% (E1). The extraction efficiencies (*EF*), defined as the ratio of the concentration of analyte determined by HPLC to the initial concentration of analyte in the sample, were higher than 98% for the three analytes.

Chromatographic parameters including capacity factor (*k*), separation factor (*α*), efficiency factor (*N*), and resolution equation (*R*) were also calculated to evaluate the methodology (Table 1). Reproducible retention times were observed throughout a normal working day (8–12 h of analysis).

Water samples, including wastewater and tap and lake water were analyzed to evaluate the applicability and accuracy of the proposed method on real samples (Table 2). Evaluation of results showed that concentrations of the target estrogens in these samples were below limits of detection (LODs). Samples were then spiked with E1, E2, EE2, and E3 at two different concentrations, i.e., 5 and 20 μg L^−1^, and recoveries were in the range of 88.5–99.6% and 88.4–99.9%, respectively (Table 2). Additionally, analytes did not show a significant shift in retention time and/or sensitivity in the chromatographic analysis.

In Table 3, a comparison of the proposed methodology with other reported methods that employ the same detector for estrogen determination is presented. Due to low solvent consumption (0.1 mL methanol) and selection of IL as organic phase (26 mg), which avoids solvent volatilization into the environment, the proposed method is in good agreement with green chemistry principles. In comparison with solid-phase-based microextraction techniques, the IL-on SBME provides comparable or lower extraction times. Furthermore, in comparison with DLLME-based methodologies, the use of magnetic counter-anions simplifies the extraction since formation of emulsions and centrifugation step are avoided. Evaluation of the analytical performance of this methodology indicates good repeatability and detection limits, which are comparable to most previously reported extraction procedures.

## 3. Materials and Methods

Reagents: Trihexyl(tetradecyl)phosphonium chloride ([P_66614_]Cl, 95%), potassium ferricyanide (K_3_[Fe(CN)_6_], 99.5%), iron(III) chloride hexahydrate (FeCl_3_.6H_2_O, ≥99.9%), dysprosium(III) oxide (Dy_2_O_3_, 99.9%), perchloric acid (HClO_4_, 70%), estrone (E1), 17β-estradiol (E2), estriol (E3), and 17α-ethinylestradiol (EE2) were purchased from Sigma-Aldrich (Milwaukee, WI, USA) and used without further purification. Methanol and acetonitrile were of chromatographic, anhydrous grade (Sigma-Aldrich), while water and other organic solvents were of HPLC grade (J. T. Baker, Phillipsburg, NJ, USA). Ultrapure water (18 MΩ cm) was obtained using a Millipore Continental Water System (Bedford, MA, USA).

A 0.85 g L^−1^ stock standard solution of each hormone, i.e., E3, E2, EE2, and E1 was prepared by dissolving an appropriate amount into methanol. Stock solutions were stored at 4 °C. Mixed standard stock solutions containing estrogens were prepared similarly in methanol and stored at 4 °C. Lower concentrations were prepared by diluting the stock solution with methanol. Working solutions were prepared daily by diluting standard stock solutions with deionized (DI) water. Absorbance spectra of stock solutions (background solvent mixture: water/acetonitrile (40:60)) were measured in the wavelength range of 200 to 600 nm using a Lambda 750 UV–VIS spectrometer (Perkin Elmer, Shelton, CT, USA), equipped with a 1 cm quartz cuvette.

Synthesis of the MILs: The MIL trihexyl(tetradecyl)phosphonium tetrachloroferrate(III) ([P_66614_][FeCl_4_]) was synthesized according to a previously reported procedure [11]. Briefly, FeCl_3_.6H_2_O was mixed with P_66614_Cl in a 1:1 molar ratio after both salts were dissolved in anhydrous methanol. The resultant solution was stirred at room temperature for 24 h. After complete reaction, methanol was removed using a rotavapor. The resultant liquid was washed with distilled water and then removed from the hydrophobic MIL using a Pasteur pipet. The MIL was freeze-dried overnight using a lyophilizer to remove residual traces of water. Elemental analysis: calculated for C_32_H_68_Cl_4_FeP: C, 56.40; H, 10.06; Cl, 20.81; Fe, 8.19; P, 4.24%. Found: C, 56.46; H, 10.01%. The Cl, Fe, and P concentrations were determined using inductively coupled plasma mass spectrometry (ICP–MS) as 20.75, 8.21, and 4.48%, respectively, which were in good agreement with calculated concentrations.

Trihexyl(tetradecyl)phosphonium ferricyanide ([P_66614_]_3_[Fe(CN)_6_]) was synthesized using an anion-exchange reaction between [P_66614_]Cl and K_3_[Fe(CN)_6_]. Briefly, a solution of P_66614_Cl in dichloromethane (DCM) and a supersaturated aqueous solution of K_3_[Fe(CN)_6_] were mixed at a 3:1.5 ratio. After 48 h stirring, the water phase was removed using a Pasteur pipet and the organic phase was washed with deionized water to remove the potassium chloride by-product. Finally, the product [P_66614_]_3_[Fe(CN)_6_] was dried under vacuum. Elemental analysis: calculated for C_102_H_204_N_6_FeP_3_: C, 73.64; H, 12.36; N, 5.05; Fe, 3.36; P, 5.59%. Found: C, 73.55; H, 12.17; N, 4.97%. The P and Fe concentrations were determined using ICP–MS as 5.51 and 3.28%, respectively, which were in good agreement with the calculated concentrations.

Trihexyl(tetradecyl)phosphonium dysprosium thiocyanate ([P_66614_]_5_[Dy(SCN)_8_]) was synthesized by reacting trihexyl(tetradecyl)phosphonium thiocyanate ([P_66614_][SCN]) with dysprosium perchlorate hexahydrate (Dy(ClO_4_)_3_.6H_2_O), according to a reported procedure [28]. Briefly, P_66614_Cl was mixed with KSCN in a 1:2 molar ratio after both salts were dissolved in dry acetonitrile. The reaction mixture was stirred for 2 days. After filtration, the solution was dried under vacuum. Cold DCM was then added to the mixture to dissolve the product and precipitate out the extra KSCN, which was removed by filtering. Subsequently, the product, [P_66614_][SCN], was dried under vacuum. Dy(ClO_4_)_3_·6H_2_O was obtained by dissolving Dy_2_O_3_ in 60% aqueous HClO_4_ in a 1:1 molar ratio, and subsequent removal of the excess water under vacuum. Finally, [P_66614_][SCN], KSCN, and Dy(ClO_4_)_3_·6H_2_O were mixed at a molar ratio of 5:3:1 and stirred (24 h) in dry acetonitrile to obtain [P_66614_]_5_[Dy(SCN)_8_]. During stirring, most of the KClO_4_ precipitated. To remove the remaining KClO_4_, the procedure described above to remove excess salt was applied. The final product, [P_66614_]_5_[Dy(SCN)_8_], was then dried under vacuum. Finally, a light orange liquid was obtained. Elemental analysis: calculated for C_168_H_340_N_8_DyS_8_P_5_: C, 66.24; H, 11.25; N, 3.68; Dy, 5.33; P, 5.08; S, 8.42%. Found: C, 66.17; H, 11.15; N, 3.68%. The P and Dy concentrations were determined using ICP–MS as 5.00 and 5.27%, respectively, which were in good agreement with calculated concentrations.

Viscosity of [P_66614_]_3_[Fe(CN)_6_] and [P_66614_]_5_[Dy(SCN)_8_]: Viscosity of the MILs at room temperature was measured using a TA Instruments rheometer (AR1000, New Castle, DE, USA). Plate temperature was maintained at 25 °C, and the plate geometry was used with a gap of 300 μm. In the first measurement, the viscosity was measured at constant shear rates (0.02, 0.1, 0.5, 1.0, 10, 25, 50, and 100 s^−1^). Each shear rate was held for 20 s and the measurement was averaged over 2 s intervals. In the second test, the shear rate was ramped from 0 to 100 s^−1^, over 300 s time intervals. In each test, samples were loaded and allowed to thermally equilibrate for 15 s before testing.

Determination of octanol/water partition coefficient: MILs at several concentrations (0.5–1.5 mmol L^−1^) were dissolved in water-saturated octanol. The highest peak as determined using the UV–VIS spectrum of the solution was chosen to build a calibration curve. A 1:1 (*v*/*v*) ratio of water saturated with octanol and octanol saturated with water was used to partition the MILs. Afterwards, the solution was stirred for 2 to 4 h. Tubes were then centrifuged and separated at 25 °C. MILs concentrations in the octanol phase were measured using UV–VIS spectrometry.

The equation K_(o/w)_ = [MIL]_o,e_/[MIL]_w,e_ was used to calculate the partition coefficient, where [MIL]_o,e_ and [MIL]_w,e_ represent respective MIL concentrations in octanol and water phases at equilibrium. MIL concentration in the water phase at equilibrium was calculated as the difference between initial MIL concentration in the octanol phase and the final MIL concentration in the octanol phase, i.e., after water addition and stirring.

Sample collection and conditioning: For tap water sample collection, domestic water was allowed to run for 20 min, and approximately 1000 mL was collected in a beaker. Tap water samples were analyzed immediately after sampling. Lake water samples were collected in Pyrex borosilicate amber glass containers after rinsing 3 times with water sample before collection. A sample volume of 1000 mL was collected at a depth of 5 cm below the surface. Wastewater samples were acquired from the local wastewater treatment plant (Baton Rouge, LA, USA). Once received, lake water and wastewater samples were immediately filtered through 0.45 μm pore size membrane filters (Millipore Corporation, Bedford, MA, USA) due to high concentrations of TDS (total dissolved solids) in these samples. All samples were stored at 4 °C in brown glass bottles (Nalgene; Nalge, Rochester, NY, USA) and analyzed as soon as possible.

Magnetic-based microextraction procedure: In a capped glass vial (20 mL), 26.0 ± 1.0 mg [P_66614_]_5_[Fe(CN)_6_] was placed onto a stir bar (10 × 3 mm). Then, a 15 mL aqueous sample was added with a final concentration of 0.4 g L^−1^ acetate-acetic acid buffer (pH 4.5) and 5.9% (*w*/*v*) NaCl. Due to strong paramagnetism and relatively high viscosity of [P_66614_]_5_[Fe(CN)_6_], this IL remained attached to the stir bar during stirring time (considered as extraction time), as can be observed in Figure 1a. After extraction (60 min, 300 rpm), the aqueous phase was discarded, the MIL phase was dissolved with methanol (0.1 mL), and estrogens were determined by HPLC–UV. Extraction conditions are summarized in Table 4.

HPLC analysis: Separation and quantitative analyses of estrogens in aqueous solutions were performed with a Shimadzu HPLC system (Kyoto, Japan) consisting of an SCL-10A system controller, two LC-10AD pumps, a DGU-14A degasser, a SIL-10AD autosampler, and an SPD-10AV UV–VIS detector (λ = 200 nm). Separation of analytes was performed at room temperature on a Phenomenex Luna C18 column, 100 Å pore size, 4 µm particle size, 250 mm × 4.6 mm i.d. column containing a guard column (Phenomenex, Torrance, CA, USA). Analytes were eluted using a gradient program at a flow rate of 0.5 mL min^−1^ with water (solvent A) and acetonitrile (solvent B). After 5 min of an isocratic run (70% A and 30% B), solvent A was decreased linearly (increasing B) and reached 20% (80% B) at 35 min. The column was then cleaned with 100% B for 5 min. After acquisition, 10 min post time was set for column equilibration at the initial solvent composition. Sample injection volume was 20 µL. Instrumental conditions are summarized in Table 4.

## 4. Conclusions

In this study, properties of different MILs were explored in combination with microextraction techniques for determination of estrogens. The extraction methods evaluated used magnetic and viscosity properties of the MILs. The MIL [P_66614_]_3_[Fe(CN)_6_] was selected for extraction of estrogens due to its higher hydrophobicity, lower cost of starting components, and simpler synthesis. Extraction recoveries were significantly influenced by pH of the aqueous phase, salt addition, properties of ILs, extraction time, and stirring rate. Moreover, extraction recovery varied depending on the MIL selected under the same conditions. A multivariate approach for optimization allowed successful determination of optimal microextraction conditions, resulting in LODs of 0.2–0.5 µL and low solvent consumption (lower than 0.13 mL solvent per 15 mL sample). This novel, simple, and low-cost approach for determination of estrogens was then used for analyses of several types of water samples, including wastewater, with recoveries in the range of 88.4–99.9%. Although the optimized extraction technique was combined with UV detection, other more sensitive techniques, such as mass spectrometry or fluorescence, can be used in combination with the developed microextraction technique to decrease the limit of detection of the methodology.

All in all, the present study confirms the great potential of MILs for extractions, separations, and preconcentration of organic analytes. In addition to properties such as hydrophobicity and viscosity, optimization of the MIL could be performed to increase extraction efficiency through, for example, the interaction between the IL cation and the analyte, or to monitor the faith of the MIL through fluorescent properties of some of the MILs. The design of the MIL reported here allows considerable versatility that has rarely been explored in the analytical field, and this study demonstrates the considerable potential of this approach.

## Figures and Tables

**Table 1:** Analytical parameters obtained with the proposed method.

Parameter	E3		E2		EE2		E1
Calibration range (µg L^−1^)	0.5–1000		0.5–1000		1–1000		1–1000
Correlation coefficients (r)	0.9986		0.9988		0.9990		0.9983
LOD (µg L^−1^)	0.2		0.2		0.3		0.5
RSD (%)	4.1		4.1		4.9		5.0
Retention time (min)	14.9		26.5		28.2		29.4
Capacity factor (*k’*)	2.69		5.55		5.98		6.27
Efficiency factor (*N*)	5929		17,838		9864		28,873
Separation factor (*α*)		2.06		1.08		1.05	
Resolution (*R*)		14.8		1.76		1.40	

**Table 2:** Determination of estrogens in water samples (95% confidence interval; *n* = 3).

Analyte		Tap Water		Lake Water		Wastewater	
	Added (µg L^−1^)	Found (µg L^−1^)	Recovery (%)	Found (µg L^−1^)	Recovery (%)	Found (µg L^−1^)	Recovery (%)
E1	5.00	4.98 ± 0.25	99.6	4.89 ± 0.24	97.8	4.53 ± 0.23	90.6
	20.0	19.6 ± 0.88	98.1	19.3 ± 0.87	96.5	17.9 ± 0.72	89.5
E2	5.00	4.92 ± 0.24	98.3	4.83 ± 0.22	96.6	4.43 ± 0.21	88.5
	20.0	19.7 ± 0.85	98.6	19.6 ± 0.95	97.9	17.8 ± 0.86	89.2
EE2	5.00	4.93 ± 0.20	98.5	4.91 ± 0.20	98.1	4.45 ± 0.25	88.9
	20.0	20.0 ± 0.81	99.9	19.5 ± 0.82	97.8	17.7 ± 0.74	88.4
E3	5.00	4.93 ± 0.21	98.6	4.90 ± 0.19	98.0	4.50 ± 0.19	89.9
	20.0	19.8 ± 0.82	98.9	19.6 ± 0.81	98.2	18.1 ± 0.75	90.3

**Table 3:** Comparison of the proposed method with microextraction-based methodologies previously reported for the determination of estrogens in water samples prior to HPLC-UV.

Technique	Estrogens	Extraction Time (min)	LOD (µg L^−1^)	RSD (%)	Sample Consumption (mL)	Calibration Range (µg L^−1^)	Reference
UASEME	E1, E2, diethylstilbestrol (DES)	<15	0.1–0.2	<1.28	10	10–1000	[35]
SBSE-LD	E1, E2, EE2, DES, mestranol, progesterone, norethisterone, norgestrel	120	0.3–1.0	<17.1	30	1.25–50.0	[36]
HF-LLLME	E1, E2, E3, EE2, DES, dienestrol (DIS), bisphenol-A, 4-t-octylphenol	50	0.11–0.66	<8.4	6	0.5–500 2–1000	[37]
HF-LPME	E2	60	0.1	5.5	140	1–1000	[38]
DLLME	E1, E2, DES	<11	0.008–0.010	<4.9	5	0.020–500.0	[39]
DLLME	E1, E2, EE2	0.5	0.003–0.020	---^a^	8	0.01–0.5 (E2, EE2) 0.04–4 (E1)	[40]
IL-DLLME	E1, E2, E3, EE2, DES	<21	0.08–0.5	<5.7	5	0.2–100 1.0–100	[33]
IL-DLLME	E1, E2, EE2, DES, DIS, hexestrol	1	13.8–37.1	<8.3	---^a^	1.5–1732	[41]
IL-on SBME	E1, E2, E3, EE2	60	0.2–0.5	< 5.1	15	1.0–1000	Present work

Abbreviations: UASEME: ultrasound-assisted surfactant-enhanced emulsification microextraction; SBSE-LD: stir bar sorptive extraction with in situ derivatization and liquid desorption; HF: hollow fiber liquid-phase microextraction; HF-LLLME: HF-based liquid–liquid–liquid micro-extraction; DLLME: dispersive liquid–liquid microextraction. ^a^ Not reported.

**Table 4:** Experimental and instrumental conditions for estrogens determination.

Extraction Conditions			
Pre-treated sample volume (mL)		15	
MIL mass (mg)		26	
pH		4.5	
NaCl concentration (% (*w*/*v*))		6	
Extraction time (min)		60	
Stirring rate (rpm)		300	
HPLC Instrumental Conditions			
Selected absorption wavelength		200 nm	
Injection volume		20 µL	
LC column		Luna C18 (4 µm × 4.6 mm i.d. × 250 mm)	
Flow rate		0.5 mL min^−1^	
Column temperature		25 °C	
Mobile phases		A: water B: acetonitrile	
HPLC Gradient Program			
Step	Initial Time (min)	Final Time (min)	Final Composition of Mobile Phase
0	0.0	5.0	70% A; 30% B (isocratic)
1	5.0	35	20% A; 80% B (linear gradient)
2	35	40	100% B (isocratic)

## Data Availability

The data presented in this study are available in insert article.

## Sample Availability

Samples of the compounds are not available from the authors.

## References

[1] Ramos L. (2012). Critical overview of selected contemporary sample preparation techniques. J. Chromatogr. A.

[2] Jeannot M.A. (2007). Extraction—Liquid phase microextraction. Encycl. Sep. Sci..

[3] Kokosa J.M. (2013). Advances in solvent-microextraction techniques. TrAC-Trend Anal. Chem..

[4] Anthony J.L., Brennecke J.A., Holbrey J.D., Maginn E.J., Mantz R.A., Rogers R.D., Trulove P.C., Visser A.E., Welton T., Wasserscheid P., Welton T. (2002). Physicochemical properties of ionic liquids. Ionic Liquids in Synthesis.

[5] Trujillo-Rodriguez M.J., Nan H., Varona M., Emaus M.N., Souza I.D., Anderson J.L. (2019). Advances of ionic liquids in analytical chemistry. Anal. Chem..

[6] Fraser K.J., MacFarlane D.R. (2009). Phosphonium-based ionic liquids: An overview. Aust. J. Chem..

[7] Stojanovic A., Morgenbesser C., Kogelnig D., Krachler R., Keppler B.K., Kokorin A. (2011). Quaternary ammonium and phosphonium ionic liquids. Chemical and Environmental Engineering, Ionic Liquids: Theory, Properties, New Approaches.

[8] Berton P., Wuilloud R.G. (2011). An online ionic liquid-based microextraction system coupled to electrothermal atomic absorption spectrometry for cobalt determination in environmental samples and pharmaceutical formulations. Anal. Methods.

[9] Martinis E.M., Berton P., Altamirano J.C., Hakala U., Wuilloud R.G. (2010). Tetradecyl(trihexyl)phosphonium chloride ionic liquid single-drop microextraction for electrothermal atomic absorption spectrometric determination of lead in water samples. Talanta.

[10] Martinis E.M., Escudero L.B., Berton P., Monasterio R.P., Filippini M.F., Wuilloud R.G. (2011). Determination of inorganic selenium species in water and garlic samples with on-line ionic liquid dispersive microextraction and electrothermal atomic absorption spectrometry. Talanta.

[11] Deng N., Li M., Zhao L., Lu C., de Rooy S.L., Warner I.M. (2011). Highly efficient extraction of phenolic compounds by use of magnetic room temperature ionic liquids for environmental remediation. J. Hazard. Mater..

[12] Cruz M.M., Borges R.P., Godinho M., Marques C.S., Langa E., Ribeiro A.P.C., Lourenço M.J.V., Santos F.J.V., de Castro C.A.N., Macatrão M. (2013). Thermophysical and magnetic studies of two paramagnetic liquid salts: [C_4_mim][FeCl_4_] and [P_66614_][FeCl_4_]. Fluid Phase Equilibria.

[13] Clark K.D., Nacham O., Purslow J.A., Pierson S.A., Anderson J.L. (2016). Magnetic ionic liquids in analytical chemistry: A review. Anal. Chim. Acta.

[14] Sajid M. (2019). Magnetic ionic liquids in analytical sample preparation: A literature review. Trends Anal. Chem..

[15] Yu H., Merib J., Anderson J.L. (2016). Faster dispersive liquid-liquid microextraction methods using magnetic ionic liquids as solvents. J. Chromatogr. A.

[16] Chatzimitakos T.G., Pierson S.A., Anderson J.L., Stalikas C.D. (2018). Enhanced magnetic ionic liquid-based dispersive liquid-liquid microextraction of triazines and sulfonamides through a one-pot, pH-modulated approach. J. Chromatogr. A.

[17] Benede J.L., Anderson J.L., Chisvert A. (2018). Trace determination of volatile polycyclic aromatic hydrocarbons in natural waters by magnetic ionic liquid-based stir bar dispersive liquid microextraction. Talanta.

[18] Chisvert A., Benede J.L., Anderson J.L., Pierson S.A., Salvador A. (2017). Introducing a new and rapid microextraction approach based on magnetic ionic liquids: Stir bar dispersive liquid microextraction. Anal. Chim. Acta.

[19] Fiorentini E.F., Escudero L.B., Wuilloud R.G. (2018). Magnetic ionic liquid-based dispersive liquid-liquid microextraction technique for preconcentration and ultra-trace determination of Cd in honey. Anal. Bioanal. Chem..

[20] Fiorentini E.F., Canizo B.V., Wuilloud R.G. (2019). Determination of as in honey samples by magnetic ionic liquid-based dispersive liquid-liquid microextraction and electrothermal atomic absorption spectrometry. Talanta.

[21] Merib J., Spudeit D.A., Corazza G., Carasek E., Anderson J.L. (2018). Magnetic ionic liquids as versatile extraction phases for the rapid determination of estrogens in human urine by dispersive liquid-liquid microextraction coupled with high-performance liquid chromatography-diode array detection. Anal. Bioanal. Chem..

[22] Feng X., Xu X., Liu Z., Xue S., Zhang L. (2020). Novel functionalized magnetic ionic liquid green separation technology coupled with high performance liquid chromatography: A rapid approach for determination of estrogens in milk and cosmetics. Talanta.

[23] Chatzimitakos T.G., Anderson J.L., Stalikas C.D. (2018). Matrix solid-phase dispersion based on magnetic ionic liquids: An alternative sample preparation approach for the extraction of pesticides from vegetables. J. Chromatogr. A.

[24] Trujillo-Rodriguez M.J., Pino V., Anderson J.L. (2017). Magnetic ionic liquids as extraction solvents in vacuum headspace single-drop microextraction. Talanta.

[25] Racz L., Goel R.K. (2010). Fate and removal of estrogens in municipal wastewater. J. Environ. Monit..

[26] Ting Y.F., Praveena S.M. (2017). Sources, mechanisms, and fate of steroid estrogens in wastewater treatment plants: A mini review. Environ. Monit. Assess..

[27] Briciu R.D., Kot-Wasik A., Namiesnik J. (2009). Analytical challenges and recent advances in the determination of estrogens in water environments. J. Chromatogr. Sci..

[28] Mallick B., Balke B., Felser C., Mudring A.-V. (2008). Dysprosium room-temperature ionic liquids with strong luminescence and response to magnetic fields. Angew. Chem. Int. Ed..

[29] Xu L., Basheer C., Lee H.K. (2010). Solvent-bar microextraction of herbicides combined with non-aqueous field-amplified sample injection capillary electrophoresis. J. Chromatogr. A.

[30] Burghoff B., Goetheer E.L.V., de Haan A.B. (2008). COSMO-RS-based extractant screening for phenol extraction as model system. Ind. Eng. Chem. Res..

[31] Myers R.H., Montgomery D.C. (1995). Response Surface Methodology: Process and Product Optimization Using Designed Experiments.

[32] Derringer G.C., Suich R. (1980). Simultaneous optimization of several response variables. J. Qual. Technol..

[33] Wu C.-Q., Chen D.-Y., Feng Y.-S., Deng H.-M., Liu Y.-H., Zhou A.-J. (2012). Determination of estrogens in water samples by ionic liquid-based dispersive liquid-liquid microextraction combined with high performance liquid chromatography. Anal. Lett..

[34] Melwanki M.B., Huang S.-D. (2006). Three-phase system in solvent bar microextraction: An approach for the sample preparation of ionizable organic compounds prior to liquid chromatography. Anal. Chim. Acta.

[35] Zou Y., Li Y., Jin H., Zou D., Liu M., Yang Y. (2012). Ultrasound-assisted surfactant-enhanced emulsification microextraction combined with HPLC for the determination of estrogens in water. J. Braz. Chem. Soc..

[36] Almeida C., Nogueira J.M.F. (2006). Determination of steroid sex hormones in water and urine matrices by stir bar sorptive extraction and liquid chromatography with diode array detection. J. Pharm. Biomed..

[37] Chen B., Huang Y., He M., Hu B. (2013). Hollow fiber liquid-liquid-liquid microextraction combined with high performance liquid chromatography-ultraviolet detection for the determination of various environmental estrogens in environmental and biological samples. J. Chromatogr. A.

[38] Li H., Jiang Y., Liu Y. (2011). Enrichment and determination of trace estradiol in environmental water samples by hollow-fiber liquid-phase microextraction prior to HPLC. J. Chromatogr. Sci..

[39] Hadjmohammadi M.R., Ghoreishi S.S. (2011). Determination of estrogens in water samples using dispersive liquid liquid microextraction and high performance liquid chromatography. Acta Chim. Slov..

[40] Sousa É.M.L., Dias R.A.S., Sousa E.R., Brito N.M., Freitas A.S., Silva G.S., Silva L.K., Lima D.L., Esteves V.I., Silva G.S. (2020). Determination of three estrogens in environmental water samples using dispersive liquid-liquid microextraction by high-performance liquid chromatography and fluorescence detector. Water Air Soil Pollut..

[41] Socas-Rodríguez B., Hernández-Borges J., Asensio-Ramos M., Herrera-Herrera A.V., Palenzuela J.A., Rodríguez-Delgado M.A. (2014). Determination of estrogens in environmental water samples using 1,3-dipentylimidazolium hexafluorophosphate ionic liquid as extraction solvent in dispersive liquid–liquid microextraction. Electrophoresis.

